# Psychometric Properties of Parent Outcome Measures Used in RCTs of Antenatal and Early Years Parent Programs: A Systematic Review

**DOI:** 10.1007/s10567-019-00276-2

**Published:** 2019-02-22

**Authors:** Sarah L. Blower, Nicole Gridley, Abby Dunn, Tracey Bywater, Zoe Hindson, Maria Bryant

**Affiliations:** 1 Department of Health Sciences, University of York, York, YO10 5DD UK; 2 Clinical Trials Research Unit, Leeds Institute of Clinical Trials Research, University of Leeds, Leeds, UK; 3 Present Address: Carnegie School of Education, Leeds Beckett University, Leeds, UK

**Keywords:** Systematic review, Outcome measures, Parenting, Psychometric properties, COSMIN

## Abstract

Parenting programs are effective in the early intervention and treatment of children’s social, emotional and behavioural difficulties. However, inconsistency in the use of outcome measures limits the comparability of programs and creates challenges for practitioners seeking to monitor progress of families in their care. A systematic review was conducted to identify measures, appraise their psychometric properties and ease of implementation, with the overall objective of recommending a small battery of measures for use by researchers and practitioners. This article provides an overview of the most commonly used measures in experimental evaluations of parenting programs delivered to parents of children up to, and including, the age of 5 years (including antenatal programs). An in-depth appraisal of the psychometric properties and ease of implementation of parent outcome measures is also presented (findings in relation to child and dyadic outcome measures are presented elsewhere). Following a systematic search, 64 measures were identified as being used in three or more of 279 included evaluation studies. Data on the psychometric properties of 18 parent outcome measures were synthesised from 87 development and validation studies. Whilst it was not possible to identify a definitive battery of recommended measures, we are able to recommend specific measures that could be prioritised in further research and development and hold promise for those seeking to monitor the outcomes of parents and children in receipt of parenting programs.

**Electronic supplementary material:**

The online version of this article (10.1007/s10567-019-00276-2) contains supplementary material, which is available to authorized users.

Due to the high prevalence of social, emotional and behavioural difficulties in children, their negative long-term sequelae and associated service costs, early intervention and prevention has been identified as a key public health priority (Jones et al. 2015). Parenting programs are an effective approach for promoting child social, emotional and behavioural development (Ryan et al. 2017); however, further research is needed to establish effectiveness in the early years. The lack of consistency in measures used across parent program research studies and in routine service monitoring and evaluation, and a lack of synthesised information on the validity and reliability of measures for the 0–5 age range, hamper both researchers and practitioners seeking to establish the effectiveness of parenting programs.

Systematic reviews report that targeted group-based programs for parents of children aged 3 years and older positively impact on child behaviour and symptoms of conduct disorder (Barlow et al. 2014; Furlong et al. 2012). However, further research is needed, in the 0–3-year-old age range, to include antenatal support (Barlow et al. 2010). Evidence-based parenting programs (EBPPs) include (but are not limited to) Incredible Years (Webster-Stratton and Reid 2003), Parent–Child Interaction Therapy (Brinkmeyer and Eyberg 2003) and Triple P (Sanders 1999). The content of parenting programs may differ, yet many incorporate the general principles of social learning theory, attachment theory and cognitive-behavioural approaches (Barlow et al. 2016). These theoretical approaches emphasise the role of caregivers in shaping child socialisation, parent–child bonding and parenting practices, respectively. Parenting programs have demonstrated positive effects for parents, including reductions in maternal depression and improvements in parental wellbeing and other parental psychosocial outcomes (Hutchings et al. 2012).

Changes in parent behaviours, attitudes, skills, practices and mental health impact on child outcomes; however, *different* measures to assess such outcomes are sometimes used to measure the same constructs (Wolpert et al. 2016). This level of inconsistency across research, and also practice, is problematic because: not all measures show the same degree of improvement in parent and child functioning as a result of parent training (Patterson and Forgatch 1995); it limits the comparability of program effectiveness and cost-effectiveness and it may subsequently bias decision-making in children’s services policy and practice.

When selecting measures, validity and reliability is key concern. Validity is defined as the degree to which an instrument measures the construct(s) it purports to measure (de Vet et al. 2015). The three specific types of validity are (1) *content validity*—the degree to which a measure is an adequate reflection of the construct that it intends to measure usually determined by agreement amongst experts; (2) *construct validity*—the degree to which the scores of an instrument are consistent with hypotheses, e.g. in relation to internal relationships, scores on other instruments or differences between relevant groups and (3) *criterion validity*—the degree to which scores of a measure are an adequate reflection of the gold standard. Reliability is the degree to which a measure is free from measurement error and covers measurement properties such as *internal consistency* (the degree of interrelatedness among items), *test–retest reliability* (stability in scores over time), *inter-rater reliability* (relationship between scores from different people at the same time), *intra-rater reliability* (relationship between scores from the same person at different times) (de Vet et al. 2015).

A range of parent self-report questionnaires, observation tools, interview schedules and standardised developmental assessments have been investigated in systematic and non-systematic reviews of parenting measures (e.g. Hurley et al. 2014; Deighton et al. 2014; Wittkowski et al. 2017). These reviews have provided critical information about relevant outcomes measures, yet evidence gaps remain. First (to our knowledge), no systematic reviews of parent outcomes have exclusively focused on the antenatal to 5-year age range. The identification of this evidence gap is crucial given the prevention and early intervention agenda. Second, parenting programs achieve their impact on numerous child outcomes via a range of mediators and moderators (Gardner et al. 2010) but many systematic reviews are constrained to measures of one specific outcome (e.g. Wittkowski et al. 2017). While there are often logical and pragmatic reasons for a narrow focus, this makes it difficult for researchers, and especially practitioners, to select robust measurement tools in instances where multiple outcomes are expected. Third, few studies have considered (and accounted for) the methodological quality of validation papers in their findings, making it impossible to determine the strength of the evidence for measures, and how much confidence to place in reported validity and reliability. For example, in a review of parenting measures, Hurley et al. (2014) distinguished between measures with many validation studies and those with a small number of validation studies, allowing readers to weigh the evidence according to this metric, with the implication that a greater number of studies reflected increased confidence. Fourth, previous reviews of measurement properties have not considered implementation factors such as cost, user-friendliness, time to complete/administer a measure and availability (i.e. can the measure be accessed, and at what cost). Researchers and practitioners need *practical* measures for real-world contexts.

Selecting measures involve balancing psychometric properties, feasibility of implementation, acceptability amongst parents and alignment with common program objectives (Wolpert et al. 2016). Without quality evidence to inform measure choice, researchers and practitioners may make arbitrary, or potentially inappropriate, selections (Windle et al. 2011). A comprehensive review of the psychometric properties of measures of a range of potential primary and secondary outcomes arising from parent programs for parents of children (in the antenatal stage and up to and including age 5 years), balanced with practical implementation factors/considerations, is therefore needed.

## The Current Study

The main aim of the current study was to develop a small battery of recommended measures for both researchers and practitioners involved in the evaluation, or monitoring, of parenting programs delivered in the early years. The battery was intended to comprise measures with robust measurement properties drawn from those *most commonly used* in previous randomised controlled trials of parenting programs (with the expectation that such rigorous trials would administer the most appropriate and robust measures) and selected with consideration of factors affecting ease of implementation. The specific research questions (RQ) were as follows: (1) What measures are used in randomised controlled trials (RCTs) to evaluate outcomes of parenting programs delivered antenatally and/or for parents with children aged up to 5 years? (2) What are the measurement properties of the identified outcome measures? (3) What are the implementation properties of the measures and what factors might influence their acceptability among key stakeholders?

Systematic reviews address RQ1 and RQ2. A qualitative consultation exercise with parents and practitioners addresses RQ3. Due to the size and scope of the systematic review, and the large number of questionnaires and observational tools found, findings are reported in three review articles. This study presents the overarching rationale and methodology for the study and findings in relation to RQ1. Findings specifically relating to *parent outcome* measures for RQ2 are also presented. Child outcome measures reviewed in response to RQ2 are reported in the second review (Gridley et al. 2019a), and the results of our appraisal of dyadic (parent–child relationship) outcome measures are presented in the third review (Gridley et al. 2019b). This study was registered with PROSPERO, an international database of prospectively registered systematic reviews in health and social care housed by the University of York’s Centre for Reviews and Dissemination (CRD). PROSPERO Registration number: CRD42016039600.

## Method

The systematic review in response to RQ1 and RQ2 comprised a two-stage search process. Search 1 related specifically to RQ1, and sought to identify measures (questionnaires, developmental tests and observational tools) used to assess or measure change following attendance on a parenting program, evaluated in a RCT and published in the scientific literature. Search 2 relates to RQ2 and comprises a targeted article search on development and/or testing of measures identified (in three or more RCTs) following search 1.

Prior to the systematic review, a brief mapping exercise was undertaken by two researchers (SB and TB) to define the outcome domains. The mapping exercise results were peer reviewed via the parenting steering group in the Healthy Child and Healthy Families theme of the Collaboration in National Institute for Health Research Collaboration for Leadership in Applied Health Research and Care Yorkshire and Humber (NIHR CLAHRC-YH). Outcome domains were mapped under three categories representing the population of interest and included parent outcomes (parenting skills and practices, parenting attitudes and beliefs [including confidence], depression and general psychological wellbeing), child (social and emotional development/wellbeing, cognitive development), behaviour (social and antisocial) and dyadic outcomes (interaction, attachment, bond and maternal sensitivity).

### Measures Used in RCTS of Parenting Programs (Search 1)

#### Search Strategy (Search 1)

**Fig. 1:**
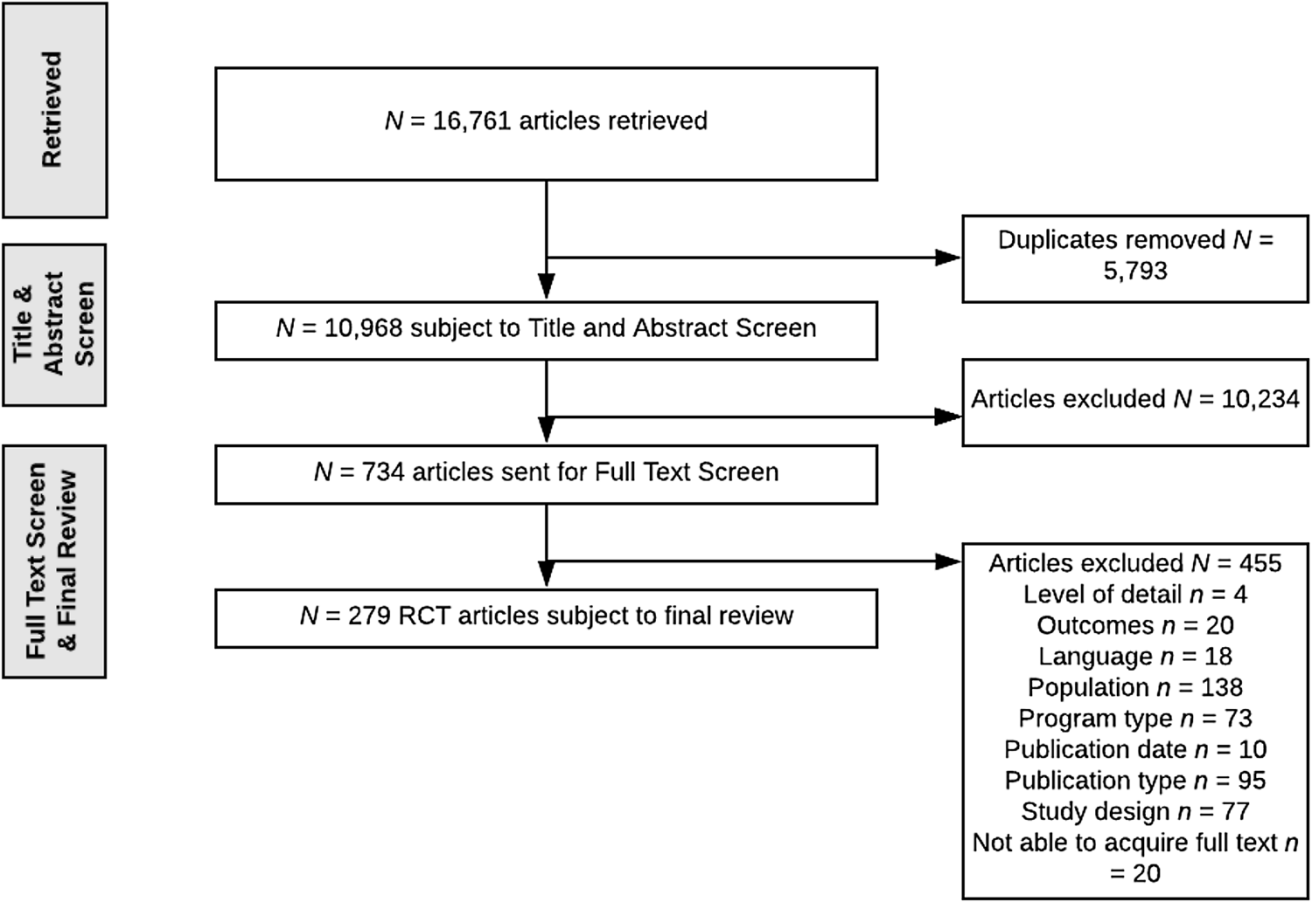
Flowchart of article retrievals for Search 1

A range of social science, biomedical and health economics databases were searched: EBSCO (CINAHL plus [1991–2015]; ERIC); OVID (PsycINFO [1987 to June week 5 2015]; PsycARTICLES [full text]; EconLit [1886 to June 2015]; Maternity and Infant care Database [MIDIRS]; Social Policy and Practice database [SOPP]; EMBASE [1980–2015]); Web of science core collection (Social Science Citation Index expanded; Social Sciences Citation Index; Arts and Humanities Citation Index; Conference Proceedings Index); ProQuest (ASSIA; British Nursing Index [available from 1996]); OVID (MEDLINE Journal articles; OVID medline 1946 to May week 4 2015, OVID medline without revisions 1996 to May week 4 2015 and OVID medline daily update May 28, 2015); Centre for Reviews and Dissemination (DARE, HTA, NHS EED) and the Cochrane Library. An example of the search strategy for retrieving relevant RCT evaluations is available as Electronic Supplementary Material (ESM). The search was limited to English language publications. See Fig. 1 for flowchart of article retrievals.

### Inclusion and Exclusion Criteria (Search 1)

Search 1 focused specifically on identifying parent, child or dyadic outcome measures used in ‘high-quality’ parent program evaluations, i.e. RCTs (the perceived gold standard design). Inclusion criteria (restricted only to peer-reviewed items) were manuscripts including the following: (1) Primary research relating to the evaluation of the effectiveness of a parenting program using RCT methodology with a ‘treatment’ and ‘comparison group’ (any comparator, e.g. control, waiting list, other treatments) with participants randomly allocated to groups; (2) Samples that included expectant parents, mothers and/or fathers or other types of primary carer, of children up to and including the age of 5 years; (3) A parent program that met our definition (i.e. structured, manualised, delivered over three or more sessions by trained facilitator and designed to improve some aspect of children’s social and emotional wellbeing or behaviour and to include antenatal programs); (4) At least one relevant outcome measured using an independently developed measure (i.e. a general measure not specifically designed to measure the intervention under evaluation); (5) A study written in the English language published between 1995 and 2015.

Exclusion criteria were as follows: (1) Papers with insufficient/missing information in the full text to determine eligibility. (2) The manuscript was not available to download in full text format via institutional subscriptions.

Retrieved articles were downloaded into an Endnote database and duplicate articles were removed. Three reviewers (SB, NG and ZH) independently performed the eligibility assessment of the remaining articles initially via a title and abstract screen and followed by a full text screen. Inter-rater reliability checks were performed on a 20% random selection of all identified and included articles, and a 20% random selection of all excluded articles. There were no recorded disagreements between reviewers.

### Data Extraction and Synthesis (Search 1)

Three reviewers (SB, NG and KT) independently extracted data from the remaining articles into a data extraction form. Following data extraction, two reviewers (SB and NG) performed data synthesis to identify a list of all the measures and the frequency of their use across the included studies. The measures were then grouped according to their administration format, i.e. questionnaires, developmental tests or observational tools. In order to create a definitive list of measures for psychometric property and ease of implementation appraisal, an eligibility assessment was performed. To ensure that the final review included the most commonly used measures, a threshold of three or more independent occurrences in the included (RCT) studies was applied. Other measure inclusion criteria included that it was a quantitative measure; the shortest (and most recent) version; administered in the English language and measured either parent, child or dyadic outcomes.

### Development and Validation Studies of Identified Measures (Search 2)

Search 2 was designed to retrieve all relevant development and validation studies for the measures identified for appraisal following Search 1.

#### Search Strategy (Search 2)

Databases were as for Search 1, but excluded Centre for Reviews and Dissemination (DARE, HTA, NHS EED) and the Cochrane Library. No limitations on publication year were used (we used the first allowable search dates through to November 2016). Searches were limited to English language. It can be difficult to identify papers reporting the development or validation of outcome measures due to a lack of consistency in the use of indexing, and keywords used by different databases (Bryant et al. 2014). Subsequently, this review utilised a complex key search term syntax developed by Terwee et al. (2009) which, firstly facilitates the comparison of the current findings with previous work in this area. Secondly, during initial pilot searches, the complex search term produced fewer returns from each database yet these returns were more likely to meet the eligibility criteria for review. See ESM for an example of the search strategy.

#### Eligibility Criteria (Search 2)

Inclusion criteria were that the article (1) described the development or validation of a measure identified in Search 1; (2) reported on a sample of expectant parents, mothers and/or fathers and other types of primary carer, of children up to and including the age of 5 years; (3) was published in the English language and (4) was published as full text original article and available via research team’s institutional subscriptions. Exclusion criteria for retrieved articles were the opposite of the above plus (1) the focus of the manuscript was to compare different measures, or properties for the purposes of diagnostic assessment or screening, and not monitoring or measuring an outcome and (2) the sample comprised exclusively of clinical subpopulations (e.g. autism, learning disabilities, cancer patients).

Retrieved articles were downloaded into an Endnote database and duplicates removed. Three reviewers (SB, NG and AD) independently assessed the eligibility by performing an initial title and abstract screen followed by a full text screen. Prior to data extraction, inter-rater reliability checks were performed on a 20% random selection of articles for each tool included in the review, and a random 20% selection of articles excluded at the full text screen stage. Approximately 1% of all articles resulted in a disagreement between researcher dyads (either SB and NG; SB and AD or NG and AD). Disagreements were resolved via consultation with the third reviewer.

### Data Extraction and Synthesis (Search 2)

Data were extracted from eligible articles on pre-prepared data extraction forms using Qualtrics software, and structured in accordance with two appraisal checklists: (1) the COnsensus-based Standards for the selection of health Measurement INstruments (COSMIN; Terwee et al. 2011a) checklist and (2) the Terwee et al. (2011b) quality criteria for measurement properties checklist. Inter-rater reliability tests were performed on 100% of all extracted data and resolved disagreement by consensus.

The COSMIN is a 10-domain checklist rated across a four-point scale (i.e. poor, fair, good or excellent), which is used to rate the quality of an individual study’s methodology. For more details, see de Vet et al. (2015). Three reviewers (SB, NG and AD) independently extracted data from each article pertaining to methods used to assess the following properties (where applicable): (1) internal consistency (11 items), (2) reliability (14 items), (3) measurement error (11 items), (4) content validity (5 items), (5) structural validity (7 items), (6) hypothesis testing (10 items), (7) cross-cultural validity (15 items), (8) criterion validity (7 items), (9) responsiveness (18 items) and (10) interpretability (7 items). A rating was assigned to represent the methodological quality of a study investigating these properties by taking the lowest score of any item within that property (i.e. excellent, good, fair or poor).

Following completion of the COSMIN checklist, an assessment of the quality of the psychometric evidence was performed using the Terwee et al. (2011b) checklist. This checklist can be used alongside the COSMIN tool to provide a rating of the evidence of each domain on a three-point scale (positive [+], indeterminate [?] or negative [−]). Prior to data extraction, modifications to this system were made to ensure that it met the specifications of the current review. The modified checklist (available in ESM) incorporated components drawn from similar systems employed by Heinl et al. (2016), Terwee et al. (2007) and De Vet et al. (2015). Score sheets were developed in Excel to summarise the methodological quality and findings of each study. Criteria set out in the COSMIN checklist were applied to synthesise the findings for each of the measures by measurement property.

## Results

### Measures Used in RCTS of Parenting Programs (Search 1)

Search 1 resulted in the retrieval of 16,761 articles, ultimately 279 articles were subject to data extraction (see Fig. 1). The 279 articles comprised peer-reviewed and published RCT evaluations of 113 parenting programs. The programs included a variety of clinic and community based one-to-one programs (e.g. Family Check-Up, Video Feedback and Parent–Child Interaction Therapy) and group-based programs (e.g. Incredible Years and Triple P). Target populations across individual studies varied in terms of size (i.e. range *N* = 24–5563), target caregiver (e.g. mothers only or mothers and fathers), ethnicity and country of study, thus suggesting a full representation of the literature. Collectively, 480 measures were used across the 279 studies. This included questionnaires (*N* = 268), developmental tests (*N* = 55), observational tools (*N* = 106) and other formats (*N* = 51) such as clinical interview schedules. Following the application of criteria, including the frequency of use/occurrence across studies, 25 *parent* outcome measures (all questionnaires), 24 *child* outcome measures (17 questionnaires and 7 development tests) and 14 *dyadic* outcome measures (all observational tools) were identified as eligible and thus sent forward into Search 2.

### Development and Validation Studies of Identified Measures (Search 2)

The aim of Search 2 was to identify all relevant development and validation studies relating to each of the included measures. Due to the large number of measures and volume of studies identified through Search 2, this article reports on the appraisal of parent outcome measures only.

**Fig. 2:**
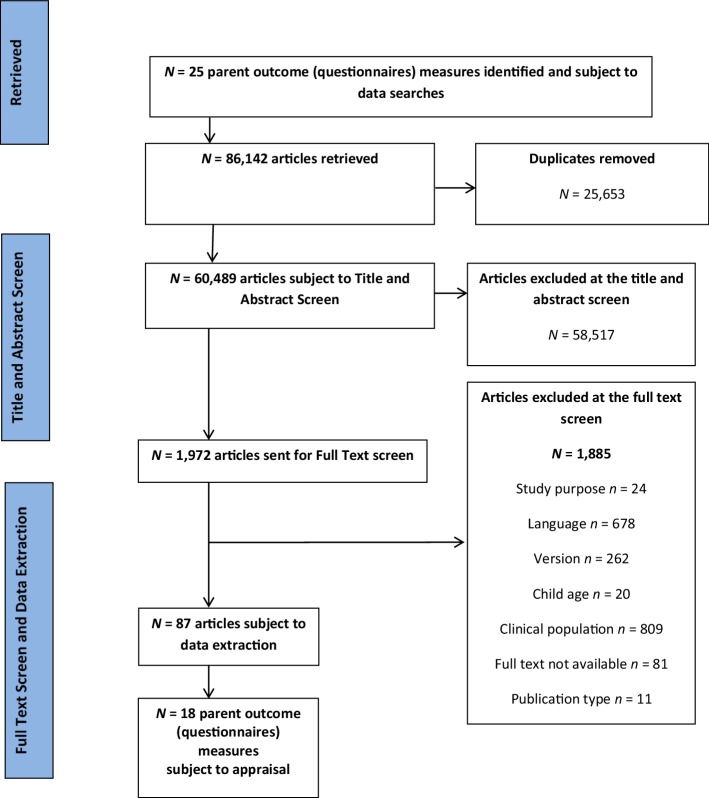
Flowchart of article retrievals for the 18 parent outcome measures reviewed

Search 2 resulted in the retrieval of 86,142 articles relating to 25 parent outcome measures, ultimately leading to the inclusion of 87 eligible articles describing the validation or development of 18 questionnaires measuring a variety of parent outcomes (Fig. 2). Seven of the original 25 measures were excluded as development or validation studies were not retrieved during the search. A description of the 18 included measures can be found in Table 1.

**Table 1:** Description of the characteristics of the parent outcome measures appraised in this review

Measure (acronym)	Respondent and target population	Number and name of (sub) scales	Total items (range of scores)	Time to complete (min)	Availability	Costs (obtained in January 2018)
Adult Adolescent Parenting Inventory 2 (AAPI-2)	Adult or adolescent parents	5 (Developmental expectations, parental level of empathy, belief in the use of corporal punishment, reversing parent–child family roles, oppressing children’s power and independence)	40 (40–200)	10–15	Available to purchase from measure publisher: http://www.nurturingparenting.com	Prices available on request from measure publisher
Alabama Parenting Questionnaire Pre-school Revision (APQ-PR)	Parents of 3–5 year olds	3 (Positive parenting, negative/inconsistent parenting, punitive parenting)	32 (32–160)	5–10	Regular version available from measure developer: https://sites01.lsu.edu/faculty/pfricklab/apq/ Items retained in pre-school revision available in Clerkin et al. (2007)	Free (but copyrighted and developer requests a copy of any publications arising from use)
Beck Depression Inventory-2 (BDI-II)	Adults and adolescents (+ 13 years)	1	21 (0–63)	5–10	Available to purchase from measure publisher: https://www.pearsonclinical.com	Starter kit (manual and 25 paper forms) $138.25
Brief Symptom Inventory-18 (BSI-18)	Adults (+ 18 years)	3 (Somatisation, depression, anxiety)	18 (18–90)	8–10	Available to purchase from measure publisher: https://www.pearsonclinical.com	Hand-Scoring Starter Kit (Includes BSI 18 manual, 50 answer sheets with test items and 50 profile forms): $129.60
Center for Epidemiological Studies Depression Scale-revised (CES-D-R)	Adults	9 (Sadness, loss of interest, appetite, sleep, thinking/concentration, guilt, tiredness, movement, suicidal ideation)	20 (0–60)	5–10	Available from measure developer website: http://cesd-r.com/about-cesdr/	Free
Depression Anxiety Stress Scale 21 (DASS-21)	Adults (+ 17 years)	3 (Depression, anxiety, stress)	21 (0–63)	5–10	Available from measure developer website: http://www2.psy.unsw.edu.au/dass/	Free to download and use the questionnaire Manual: $55 (AUSD)
Edinburgh Postnatal Depression Scale (EPDS)	Mothers in the post-partum and antenatal period	1	10 (0–30)	5–10	Available in the public domain e.g. http://www.fresno.ucsf.edu/pediatrics/downloads/edinburghscale.pdf and in Cox et al. (1987)	Free
General Health Questionnaire-12 (GHQ-12)	Adolescents and adults	1	12 (0–60)	5–10	Available to purchase from measure publisher: https://www.gl-assessment.co.uk	User guide: £115.95 Pack of 100 forms: £84.95
Hamilton Depression Rating Scale (HAMD)	Adults	1	17 (0–52)	5–10	Available in the public domain e.g. https://www.outcometracker.org/library/HAM-D.pdf	Free
Maternal Emotional Style Questionnaire (MESQ)	Mothers	2 (Emotion coaching, Emotion dismissing)	22 (14–70)	5–10	Items described in original research article (Lagacé-Séguin and Coplan 2005)	Free
Parenting Scale (PS)	Parents of pre-school children and up to adolescence	3 (Lax discipline, Over reactive discipline, Hostile discipline)	30 (30–210)	5–10	Available in the public domain e.g. http://www.pti-sf.org/yahoo_site_admin/assets/docs/PS_English.242164902.pdf	Free
Parenting Sense of Competence (PSoC)	Parents	2 (Parent satisfaction, parent self-efficacy)	17 (17–102)	5–10	Available in the public domain e.g. https://www.bristol.ac.uk/media-library/sites/sps/documents/c-change/parenting-sense-of-competence-scale.pdf	Free
Parenting Stress Index Short Form (PSI-SF)	Parents of children aged 1 month to 12 years	3 (Parental distress, Parent–child dysfunctional interaction, Difficult child)	36 (36–180)	10–15	Available to purchase from measure publisher: http://www.parinc.com	Manual: $85 Pack of 25 forms: $107
Perinatal Post-traumatic Stress Disorder Questionnaire (PPQ)	Biological mothers of infants	1	14 (0–14)	5–10	Items described in original research article, e.g. DeMier et al. (2000)	Free
Rosenberg Self-Esteem Scale (RSES)	Adults (and adolescents)	1	10 (0–30)	5	Available to download from measure developer: https://socy.umd.edu/quick-links/using-rosenberg-self-esteem-scale	Free
Short Form-12 (SF-12)	Adults (+ 16 years)	2 (Physical health composite score, Mental health composite score)	12 (0–100)	5–10	License required from measure publisher: https://campaign.optum.com/optum-outcomes.html	Costs available on request from publisher
State Trait Anxiety Inventory (STAI)	Adults (+ 16 years)	2 (state anxiety, trait anxiety)	40 (40–160)	10	License to reproduce available to download from measure publisher: http://www.mindgarden.com	Pack of 50 forms and scoring instructions: $125
Symptom Checklist 90 Revised (SCL-90-R)	Adults and adolescents (+ 13 years)	9 (Somatisation, Obsessive–compulsive, Interpersonal sensitivity, Depression, Anxiety, Hostility, Phobic anxiety, Paranoid ideation, Psychoticism)	90 (0–100)	12–15	Available to purchase from measure publisher: https://www.pearsonclinical.com	Starter kit (includes 50 forms and scoring materials): $132.85

This section presents the methodological quality and findings of studies reporting the measurement properties of 18 questionnaires, as rated using the COSMIN and the Terwee (2011b) checklists. Table 2 presents the overall ratings of measurement properties for each measure. A description of the key characteristics of each included development or validation study (including the size and ethnicity of the samples) is available in ESM.

**Table 2:** Quality of measurement properties for each parent outcome measure

Measure name (total number of studies reviewed)	Internal consistency	Test–retest reliability	Inter-rater reliability	Structural validity	Convergent/divergent validity	Discriminant/known groups	Criterion validity
AAPI (1)	**−**			+	**−**		
PSoC (3)	+++			++	**---**		
RSES (9)	+++			+++	+/**−**		
APQ pre-school (1)	++			**--**			
Parenting Scale (4)	+++	**--**	**--**	+++	**--**		
MESQ (1)	+			+	**−**		
BSI (2)	++			+++	**--**		
DASS-21 (4)	+++			**---**	++		
GHQ-12 (10)	+++			+/**−**	++		++
SCL-90 (2)	?			**--**			
SF-12 (2)	?			+			+
STAI (4)	+++	?		**---**	++	++	
PSI (4)	+/−	?		---	--		
PPQ (2)					++		
BDI-II (4)	+++			+/**−**	++	++	++
CES-D (10)	+++	--		+++	+/**−**		
EPDS (23)	+++	--		+/**−**	++	+	+++
HAMD (1)						+	

Strong level of evidence (+++ or ---): consistent findings in multiple studies (2 or more) of good methodological quality or in one study of excellent methodology quality; moderate level of evidence (++ or --): consistent findings in multiple studies (2 or more) of fair methodological quality or in one study of good methodological quality; limited level of evidence (+ or −): one study of fair methodological quality; conflicting level of evidence (+/−): conflicting findings; unknown (?): only studies of poor methodological quality—or criteria not met for + or − in majority of studies

### Measures of Parenting Attitudes and Beliefs

#### Adult Adolescent Parenting Inventory 2 (AAPI-2)

The AAPI-2 (Bavolek and Keene 1999) is a 40-item measure with five subscales measuring expectations of children, parental empathy towards children’s needs, use of corporal punishment, parent–child family roles and children’s power and independence. It is completed by adult or adolescent parents/caregivers and is available to purchase online. One validation study of this measure met criteria for appraisal in the current review (Conners et al. 2006). The study investigated internal consistency, structural validity and convergent validity, and the methods used to investigate each of these properties were judged to be of fair quality. Evidence of good *internal consistency* was found only for two of the five AAPI-2 subscales, (corporal punishment and lack of empathy subscales). The *structural validity* of the measure was acceptable and met the Terwee (2011a) criteria (Conners et al. 2006). With regards to *convergent validity*, scores on the AAPI-2 correlated in expected directions with scores on comparable measures. Although statistically significant, the size of the correlations reported did not reach the Terwee (2011a) standard required for evidence of convergent validity.

### Parenting Sense of Competence Scale (PSoC)

The PSoC (Johnston and Mash 1989) is a 17-item self-report questionnaire completed by parents/caregivers. It has two subscales measuring satisfaction and efficacy with regards to parenting roles and is freely available in the public domain. Three PSOC validation studies were appraised. Two studies reported acceptable levels of *internal consistency* using methods rated as excellent (Lovejoy et al. 2010; Rogers and Matthews 2004). The *structural validity* of the PSOC was deemed acceptable and met the Terwee (2011a) criteria in a good-quality study (Rogers and Matthews 2004). PSoC was examined for *convergent validity* in three studies (Lovejoy et al. 2010; Rogers and Matthews 2004; Karp et al. 2015), in which PSOC scores were compared to scores on a range of different parent outcome measures (e.g. RSES and the Parenting Scale). Across all of the analyses, the direction of correlations was found to be in line with hypotheses and many were statistically significant; however, the size of the correlation failed to meet the Terwee (2011a) standard. All of these papers were deemed to have good methodological quality.

### Rosenberg Self-Esteem Scale (RSES)

The RSES (Rosenberg 1989) is a 10-item measure of general self-esteem, not specific to the parenting role, it is free to use and available to download online. The RSES was developed much earlier than the other two measures in this outcome domain and nine validation studies were appraised. Acceptable levels of *internal consistency* were reported in several studies rated as having excellent methodological quality (Chao et al. 2016; Hatcher and Hall 2009; Gray-Little et al. 1997; Donnellan et al. 2016; Sinclair et al. 2010).

The *structural validity* of the RSES was investigated in all nine studies though the findings and the methodological quality of those studies varied. Exploratory factor analysis was carried out in five studies; all were judged as being of a good methodological quality. Three met the Terwee (2011a) criteria (Hatcher and Hall 2009; Donnellan et al. 2016; Sinclair et al. 2010) for positive evidence of structural validity. Confirmatory factor analysis was carried out in four studies (Corwyn 2000; Vispoel et al. 2001; Hyland et al. 2014; Donnellan et al. 2016; Sinclair et al. 2010), all of which reported findings rated good for methodological quality and met the criteria for evidence of structural validity. Thus, overall the findings suggest strong evidence of sound structural validity for the RSES. Conflicting evidence of *convergent validity* was found in four studies of varying methodological quality. One (Hatcher and Hall 2009) had good methodological quality and reported evidence of convergent validity that met the Terwee (2011a) standard. Conversely, the three remaining papers (Robinson Kurpius et al. 2008; Donnellan et al. 2016; Sinclair et al. 2010) were judged to have found poor evidence of convergent validity in studies of fair methodological quality.

### Summary

Data were only available in relation to three measurement properties (none of the included studies investigated test–retest or inter-rater reliability, for example). The PSoC and RSES appear to be supported by the strongest evidence. Both of these measures are available in the public domain and can be reproduced at no cost. They are also both brief and simple to score (see Table 2). The PSoC is more widely used having been adopted in 16 relevant RCTs with the RSES and AAPI-2 both appearing in comparatively fewer (four each) RCTs. When selecting a measure of parenting attitudes and beliefs for a specific program, it is worth bearing in mind that each of these measures assesses different aspects of parenting attitudes and beliefs.

### Measures of Parenting Practices

#### Alabama Parenting Questionnaire Pre-school Revision (APQ-PR)

The APQ-PR (Clerkin et al. 2007) is a 32-item questionnaire completed by parents/caregivers of 3–5-year-old children. It measures three subscales (positive parenting, negative/inconsistent parenting and punitive parenting). The original version of the APQ (for parents/caregivers of children and adolescents) is freely available from the measure developer; however, the items retained in the version specifically adapted for pre-schoolers are found in Clerkin et al. (2007). Only one validation study of the APQ-PR met criteria for inclusion (Clerkin et al. 2007) and was rated as having good methodological quality. In this study, acceptable levels of *internal consistency* were reported for two of the three subscales; however, the alpha for punitive parenting did not meet the Terwee (2011a) threshold.

#### The Parenting Scale (PS)

The Parenting Scale (Arnold et al. 1993) is a 30-item measure that can be obtained at no cost online. It assesses three constructs (Laxness, Overreactivity, Verbosity) from the perspective of parent/caregiver self-report. Four studies investigating the measurement properties of this instrument were appraised in this review (Arney et al. 2008; Arnold et al. 1993; Rhoades and O’Leary 2007; Lorber et al.^2014^). Strong evidence of *internal consistency* was found, i.e. positive findings reported in three studies of good, excellent and fair methodological quality (Arnold et al. 1993; Rhoades and O’Leary 2007; Lorber et al. 2014). Inter-rater reliability between mothers and fathers was assessed in one good-quality study (Lorber et al. 2014); however, the reliability findings did not meet the criterion for acceptability. The test–retest reliability of the Parenting Scale investigated in three studies also failed to meet the Terwee (2011a) criterion. Overall, while there is good evidence of internal consistency, our appraisal suggests this measure has poor reliability in the population of interest. The *structural validity* of the Parenting Scale was assessed by all four included studies. Two of them were rated as excellent quality (Lorber et al. 2014; Rhoades and O’Leary 2007) with one providing a high level of support for the structural validity of the Parenting Scale (Rhoades and O’Leary 2007). Convergent validity of this measure was also assessed by all included studies, with analyses in all four papers rated as having a fair methodological quality. Reported correlations between the comparator measures and the Parenting Scale were not large enough to meet the threshold for convergent validity evidence.

#### Maternal Emotional Styles Questionnaire (MESQ)

The MESQ (Lagacé-Séguin and Coplan 2005) is a 22-item measure of maternal emotional styles, comprising two subscales that assess ‘emotion coaching’ and ‘emotion dismissing’ parenting styles. One study was included in this review and was rated of fair methodological quality (Lagacé-Séguin and Coplan 2005). The findings suggest that the measure has acceptable levels of both *internal consistency* and *structural validity*. An analysis of convergent validity was conducted; however, correlations with the comparator instrument were not large enough to meet the acceptable threshold adopted in this review.

### Summary

Our findings suggest the strongest support for the Parenting Scale, with the structural validity of the measure revealed to be particularly robust in comparison to other measures and by objective standards. This is likely to be a useful tool for practitioners given that it is free to use, relatively straightforward to score and accessible. It is also one of the most widely used measures of all those reviewed in this study across all outcome domains. Utilised in 28 RCT studies of relevant programs, there is a strong argument for the continued use of this measure both in research and in practice settings for the purposes of monitoring of parenting program outcomes. The APQ-PR was identified in three (Search 1) evaluation studies and the MESQ in four studies.

### Measures of General Psychological Wellbeing

#### Brief Symptom Inventory-18 (BSI-18)

The BSI-18 (Derogatis 2001) is an 18-item measure of psychological distress and psychiatric disorders in adults available for purchase online. The measure comprises three subscales measuring Somatisation, Depression and Anxiety. Two validation studies were appraised in this review (Houghton et al. 2012, and; Prelow et al. 2005). Based on the analyses conducted by Houghton et al. (2012) using good-quality methods, the evidence suggests the BSI-18 has acceptable *internal consistency *levels. Overall, the BSI obtained a strong rating of *structural validity* due to one study of excellent methodological quality (Prelow et al. 2005) reporting goodness-of-fit statistics that met the thresholds adopted in this review. However, it should be noted that the second study, rated as having good methodological quality (Houghton et al. 2012), reported findings that did not meet the threshold. In this instance, the findings of the study rated as excellent are weighted more significantly in determining an overall assessment of the measurement property. One study of good quality (Prelow et al. 2005) was appraised as finding evidence of poor *convergent validity* following an analysis of scores on the BSI-18 and the RSES. While a second study reported positive findings for this property, the methodological quality of the analyses was poor and thus an overall rating of poor has been assigned to this measure (Houghton et al. 2012).

#### Depression Anxiety Stress Scale 21 (DASS-21)

The DASS-21 (Lovibond and Lovibond 1995) is a measure of the negative emotional states of depression, anxiety and stress (as represented by three subscales) and is available in the public domain. Five studies of the measurement properties of DASS-21 were included (Osman et al. 2012; Henry and Crawford 2005; Sinclair et al. 2012; Gomez et al. 2014; Ronk et al. 2013). Four studies of either good (Osman et al. 2012; Henry and Crawford 2005; Sinclair et al. 2012) or excellent (Gomez et al. 2014) methodological quality reported evidence that the DASS-21 has good *internal consistency*. Three studies provided analyses of *structural validity*, while one study of good methodological quality did report statistics on structural validity that met our threshold (Osman et al. 2012), two other studies rated as either good (Sinclair et al. 2012) or excellent quality (Gomez et al. 2014) did not. The DASS-21 therefore appears to have poor structural validity. Data on *convergent validity* were presented in three studies (Osman et al. 2012; Henry and Crawford 2005; Sinclair et al. 2012). Scores on the DASS-21 were correlated in expected directions with scores on comparable instruments and with a magnitude that met our threshold for positive evidence. The methodological quality of Osman et al., (2012) and Henry and Crawford (2005) was rated as fair, with Sinclair et al. (2012) deemed good. Overall, there is a moderate level of evidence in support of the convergent validity of the DASS-21.

#### General Health Questionnaire-12 (GHQ-12)

The GHQ-12 (Goldberg and Williams 1988) is a brief 12-item measure of minor psychiatric disorders that yields a total overall score. It is available to purchase online. Ten studies were appraised in this review (Hankins 2008a, b; Banks 1983; Kalliath et al. 2004; Martin 1999; Abubakar and Fischer 2012; Doyle et al. 2012; Graetz 1991; Hu et al. 2007; Lewis and Wessely 1990). Three studies of good (Hankins 2008b; Kalliath et al. 2004) or excellent (Martin 1999) methodological quality suggest positive evidence of *internal consistency*. Although the GHQ-12 is described by developers as yielding one overall score, several studies (Hankins 2008a, b; Kalliath et al. 2004; Martin 1999; Abubakar and Fischer 2012; Doyle et al. 2012; Graetz 1991; Hu et al. 2007) investigated the factor structure of this measure, typically hypothesising multidimensional (two or three factor) models (based on the factor structures previously reported for longer versions of the GHQ). These studies are all rated good or excellent quality; however, their findings with regard to the fit of hypothesised models varied in the extent to which they met thresholds for good *structural validity* adopted in the current review (see Table 1). Furthermore, there was a suggestion that multidimensionality resulted from items loading on the basis of negative or positive phrasing in one study (Abubaker and Fisher 2012). Given that the measure is described as unidimensional and in the context of conflicting findings, the overall rating for the structural validity of the GHQ-12 has been judged inconclusive. Evidence for the *convergent validity* of the GHQ-12 is provided in Lewis and Wessely (1990); a large correlation between scores on the GHQ-12 and the Hospital Anxiety and Depression Scale (Zigmond and Snaith 1983) was reported in a study deemed to be of good methodological quality. Furthermore, our review suggests strong support for the criterion validity of the GHQ-12. In two studies of good methodological quality (Lewis and Wessely 1990; Banks 1983), this measure displayed good levels of sensitivity and specificity against criterion measures (clinical interview schedule and present state examination, respectively, and administered by trained professionals in both studies).

#### Symptom Checklist 90 Revised (SCL-90-R)

The SCL-90-R (Derogatis 1994) is a long measure of a broad range of psychological symptoms that generates scores for nine subscales representing different clusters of systems such as depression, anxiety and paranoid ideation. It is licensed by a publisher and available to purchase online. Two studies appraising the properties of this measure were included in this review (Chapman et al. 2012; Martinez et al. 2005).

*Internal consistency* was only investigated by Martinez et al. (2005) who reported acceptable levels of internal consistency; however, this property is given an unknown rating overall due to the poor methodological quality of the study. Chapman et al. (2012) examined the *structural validity* of the SCL-90-R; however, the goodness-of-fit statistics reported did not meet the threshold for good structural validity adopted in the current review. Due to the methodological quality of this study (good), the overall rating for the structural validity of the SCL-90-R is moderately poor.

#### Short Form-12 (SF-12)

The SF-12 (Ware et al. 1995) is a brief 12-item measure of psychological wellbeing that measures health and wellbeing. There are costs associated with the use of SF-12. Two validation studies of this measure were included in our review (Forero et al. 2013; Vilagut et al. 2013). Forero et al. (2013) assessed *internal consistency* in a study rated fair in methodological quality; however, the analytical techniques were outside of the scope of the criteria in the COSMIN and Terwee (2011a) checklists, thus an indeterminate rating was applied. Forero et al. (2013) also reported positive evidence for *structural validity*; due the quality of the methodology (fair), an overall rating of limited evidence was assigned for this measurement property. Similarly, our review found limited evidence of *criterion validity*. Vilagut et al. (2013) found adequate levels of sensitivity and specificity for the SF-12 in discriminating between adults with and without depressive disorders in a study rated as having fair methodological quality.

#### State Trait Anxiety Inventory (STAI)

The STAI (Spielberger et al. 1983) is a 40-item measure of state and trait anxiety. A license to obtain and reproduce the measure is available online. We appraised four studies reporting the reliability and/or validity of this measure (Maynard et al. 2010; Hundley et al. 1998; Vigneau and Cormier 2008; Bieling et al. 1998). Strong evidence of *internal consistency* was obtained from three studies, two rated as having good methodological quality (Maynard et al. 2010; Bieling et al. 1998) and the third rated excellent (Vigneau and Cormier 2008). Although analyses reported in Hundley et al. (1998) suggest poor *test–retest reliability* (by Terwee 2011a standards), the methodological quality of this study is rated as poor and thus the overall rating for this measurement property in the STAI is inconclusive. Three studies reported on the *structural validity* of the STAI with conflicting results. The first (Maynard et al. 2010) had good methodological quality and provided positive evidence of structural validity; the second study (Bieling et al. 1998), also rated as good, did not report the statistics required to apply the Terwee (2011a) criteria for good structural validity. The third study, rated as having excellent methodological quality (Vigneau and Cormier 2008), reported goodness-of-fit statistics that fall short of the Terwee (2011a) threshold. When the methodological quality of the studies is taken into consideration, the overall rating is strong evidence of poor structural validity. Two studies of *convergent validity* (Maynard et al. 2010; Bieling et al. 1998) produced a moderate level of positive evidence for this measurement property. Similarly, our appraisal supported an overall rating of moderate evidence for the discriminant validity of the STAI. This is based on one study of good methodological validity (Bieling et al. 1998) investigating differences in scores across patient subgroups comprised of those with a diagnosis of panic disorder, obsessive–compulsive disorder, social phobia or non-social phobia.

### Summary

On balance, the GHQ-12 might be considered the most promising and it was the most common measure within this outcome domain having been adopted in eight RCTs identified in Search 1 (the SF-12 and STAI are the least common appearing in three studies each). All of the measures in this category are licensed, and/or there are costs attached to their use. Another important consideration is the length of the measure, the GHQ-12 is one of the briefest containing only 12 items and requires only 5–10 min to complete (as does SF-12) when compared to, for example, the SCL-90-R, which contains 90 items.

### Measures of Parent Stress

#### The Parenting Stress Index Short Form (PSI-SF)

The PSI-SF (Abidin 1995) is a 36-item measure of parenting stress in parents/caregivers of children aged 1 month to 12 years. It generates a total score from three subscales (parental distress, parent–child dysfunctional interaction and difficult child). Four studies of the PSI-SF were included in this review (Whiteside-Mansell et al. 2007; Reitman et al. 2002; McKelvey et al. 2009; Barroso et al. 2016). All four studies reported the *internal consistency* of this measure; however, the findings are mixed and an overall rating of conflicting evidence for this property was assigned. For example, two studies, rated as having excellent methodological quality and therefore offering the strongest evidence, report differing levels of internal consistency with Whiteside-Mansell et al. (2007) offering positive evidence and McKelvey et al. (2009) reporting statistics that failed to meet the Terwee (2011a) threshold adopted in our review. The *test–retest reliabilit*y of the PSI-SF was also investigated in Barroso et al. (2016) (poor methodological quality); however, we were unable to apply the Terwee (2011a) criteria to the findings, and an overall rating of inconclusive was assigned. Three studies reported analyses of the *structural validity* of this PSI-SF using methods appraised as good (Reitman et al. 2002) and excellent quality (Whiteside-Mansell et al. 2007; McKelvey et al. 2009). A variety of different factor structures were explored in each of the studies; however, none of them reported goodness-of-fit statistics that met the threshold for our review. Overall, given the quality of the studies, our synthesis suggests strong evidence of questionable structural validity of the PSI-SF. Two studies also examined the *convergent validity* of the PSI-SF with scores on measures of theoretically linked constructs. The methodological quality of the convergent validity analyses varied from good (Whiteside-Mansell et al. 2007) to fair (McKelvey et al. 2009) and correlations with comparable measures did not meet our criteria. Thus, the overall rating for the convergent validity of the PSI-SF is moderately poor.

#### Perinatal Post-traumatic Stress Disorder Questionnaire (PPQ)

The PPQ (DeMier et al. 1996) measures post-traumatic stress symptoms associated with childbirth (including intrusiveness, avoidance and hyperarousal). It is relatively brief with only 14 items and can be obtained by compiling items from research articles. Only two validation studies (Callahan and Hynan 2002; Quinell and Hynan 1999) were eligible for inclusion in our review, both reporting levels of *convergent validity*. In both studies, correlations between PPQ and comparator measures met the threshold, both were rated of fair methodological quality and thus our appraisal resulted in an overall rating of moderate evidence for the convergent validity of the PPQ.

### Summary

Given the limited data on the psychometric properties of these two measures, it is not possible to recommend one over the other. With regards to implementation properties, the PSI-SF is a licensed measure and fees are associated with its use; whereas the PPQ is not licensed and free to use. It is also notable that the PSI-SF was included in 20 evaluation studies identified in Search 1, and the PPQ in three. This is likely due to the specific focus of the PPQ on perinatal post-traumatic stress disorder as opposed to more general stress associated with parenting roles. The selection of one of these measures over the other will therefore largely depend on the specific research and/or practice context. It is also important to note that some of the measures in the general psychological wellbeing domain contain subscales that measure stress, such as the DASS-21 which also appears to have some good evidence in support of more measurement properties than for these stress-specific measures.

### Measures of Depression

#### Beck Depression Inventory-2 (BDI-II)

BDI-II (Beck et al. 1996) is a 21-item measure of depression (suitable for use age 13 years and upwards) and is available to purchase from an online publisher. Four studies of the psychometric properties of the BDI-II were included in our review (Osman et al. 2008; Makhubela and Mashegoane 2016; Campbell et al. 2012; Kjaergaard et al. 2014). Consistent positive findings in relation to internal consistency were reported across all four studies, rated as having poor (Kjaergaard et al. 2014), fair (Osman et al. 2008) or good (Makhubela and Mashegoane 2016; Campbell et al. 2012) methodological quality. Overall, there is strong evidence in support of the *internal consistency* of this measure. Overall, we found conflicting evidence across two studies assessing the *structural validity* of the BDI-II (e.g. Osman et al. 2008; Makhubela and Mashegoane 2016). In a study of good methodological quality (Makhubela and Mashegoane 2016), the model fit statistics reported do not meet acceptable thresholds on the Terwee (2011a) rating system. In second study of fair methodological quality, Osman et al. (2008) reported positive evidence of structural validity. We found moderate evidence of acceptable levels of *convergent validity* following consistent findings in multiple studies of fair methodological quality (Osman et al. 2008; Makhubela and Mashegoane 2016; Campbell et al. 2012). Moderate evidence was also found in support of the discriminant validity of the BDI-II and provided by one study of good methodological quality (Osman et al. 2008) as was the case for criterion validity (Kjaergaard et al. 2014).

#### Center for Epidemiological Studies Depression Scale—Revised (CES-D-R)

The CES-D-R (Radloff 1977) is a relatively short instrument with 20 items measuring symptoms of depression. Scores are generated for nine subscales (sadness, loss of interest, appetite, sleep, concentration, guilt, tired, movement and suicidal ideation) and the CES-D-R is available in the public domain at no charge. Ten studies of the measurement properties of the CES-D-R were included in this review (Atkins 2014; Edwards et al. 2010; Johnson et al. 2008; Joseph and Lewis 1995; Van Lieshout et al. 2011; Maloni et al. 2005; Nguyen et al. 2004; Pretorius 1991; Orme et al. 1986 and Skorikov and Vandervoort 2003). Five studies of *internal consistency*, one of excellent methodological quality (Atkins 2014), two good quality (Pretorius 1991; Orme et al. 1986) and two of poor quality (Maloni et al. 2005 and; Skorikov and Vandervoort 2003); all provided positive evidence of internal consistency. Overall, there is strong evidence in support of this property in the CES-D-R. *Test–retest reliability* was explored in Maloni et al. (2005)—a study of good methodological quality. The findings did not meet our criteria; on the strength of this single study, the overall rating of moderate evidence of poor test–retest reliability was given. *Structural validity* was investigated in seven of the nine studies, representing the full range of possible methodological quality, e.g. poor (Orme et al. 1986), fair (Edwards et al. 2010), good (Joseph and Lewis 1995; Pretorius 1991) and excellent (Atkins 2014; Johnson et al. 2008; Van Lieshout et al. 2011; Nguyen et al. 2004). With the exception of three studies with either indeterminate (Orme et al. 1986; Pretorius 1991) or negative findings (Johnson et al. 2008), these studies reported positive evidence of structural validity. Balancing these findings against the methodological quality of each study, an overall rating of strong evidence for the structural validity of the CES-D-R was achieved. Five of the nine CES-D-R studies reported on *convergent validity*. An overall rating of conflicting evidence was determined for the convergent validity of this measure. Two studies with good and poor methodological quality reported negative findings (Atkins 2014; Pretorious 1991) and further two studies of poor quality (Maloni et al. 2005; Orme et al. 1986) and one of good quality reported positive findings (Skorikov and Vandervoort 2003).

#### Edinburgh Postnatal Depression Scale (EPDS)

The EPDS (Cox et al. 1987) is a brief measure (10 items) designed for use with mothers in the post-partum period and available in the public domain. Of all the parent measures included in this review, the largest number of validation studies was included for the EPDS (23 studies in total: Boyce et al. 1993; Carothers and Murray 1990; Chaundron et al. 2010; Cox et al. 1996, 1987; Dennis 2004; Drake et al. 2014; Edmondson et al. 2010; Harris et al. 1989; Jomeen and Martin 2007; Kernot et al. 2015; King 2012; Leverton and Elliot 2000; Logsdon et al. 2009; Matthey 2008; Matthey et al. 2013; Milgrom et al. 2005; Phillips et al. 2009; Small et al. 2007; Swalm et al. 2010; Thompson et al. 1998; Tuohy and McVey 2008 and Venkatesh et al. 2014). Specific detail on the methodological quality and findings of these studies and overall ratings are summarised here. Our data synthesis suggests strong evidence of both *internal consistency* (Drake et al. 2014; Logsdon et al. 2009; Matthey 2008; Matthey et al. 2013; Phillips et al. 2009; Small et al. 2007; Swalm et al. 2010; Tuohy and McVey 2008) and *criterion validity* (criterion measures included diagnostic interviews and assessments by trained psychologists/psychiatrists using DSM criteria; Boyce et al. 1993; Carothers and Murray 1990; Chaundron et al. 2010; Cox et al. 1996, 1987; Edmondson et al. 2010; Harris et al. 1989; Leverton and Elliot 2000; Logsdon et al. 2009; Matthey 2008; Milgrom et al. 2005; Phillips et al. 2009; Thompson et al. 1998 and Venkatesh et al. 2014). Two studies provided moderate evidence of poor *test–retest reliability* (Dennis 2004; Kernot et al. 2015). Studies suggest moderate evidence of the *convergent validity* of the EPDS with other self-report measures of depression symptoms such as the BDI and GHQ (Boyce et al. 1993; Harris et al. 1989; Logsdon et al. 2009; Matthey et al. 2013; Phillips et al. 2009; Swalm et al. 2010). Overall, there is limited but nevertheless positive evidence of *discriminant validity* for this tool (Phillips et al. 2009).

#### Hamilton Depression Rating Scale (HAMD)

The HAMD (Hamilton 1960) is a 17-item measure of depression available in the public domain. One validation study of the HAMD was included in this review. Zimmerman et al. (2013) examined the *discriminant validity* of HAMD by testing the tool’s ability to distinguish between individuals with mild, moderate and severe depression. The study had fair methodological quality and thus the positive findings reported are deemed limited evidence of the discriminant validity of HAMD overall.

### Summary

The CES-D-R is the most frequently used measure appearing in 28 evaluation studies, followed by the EPDS (15 studies), BDI-II (9 studies) and finally the HAMD (3 studies). All four measures of parental depression demonstrated positive evidence in relation to the effective functioning of one or more measurement properties. The BDI-II and EPDS provided the strongest evidence of validity and reliability in the population of interest. However, there are key differences in relation to factors associated with their implementation. The BDI-II is a licensed measure and costs are payable to the measure publisher upon use, it is also double the length of the EPDS requiring more time to complete. The EPDS was used more widely in parent program RCTs (15) than the BDI-II (in 9 studies). Both of these measures are likely to be useful for researchers and practitioners, with the EPDS focused on the postnatal period, the BDI-II is a general measure that can be used with parents/caregivers at any time, giving options for those delivering programs up to the age of 5 years.

## Discussion

This systematic review was designed to address gaps in our current understanding of the validity and reliability of a range of measures that are commonly used in the experimental evaluation of parenting programs delivered to expectant parents, or parents of children up to and including age 5 years. A key aim of the study was to support the identification of a small battery of measures, based on both measurement properties and implementation factors, that could be recommended to both researchers and practitioners in an effort to encourage more consistent use of the most robust and practical measures, and to enable comparability of programs. Search 1 revealed that RCTs use a wide variety of different measures to evaluate common outcomes of parenting programs in the antenatal period and early years. A total of 480 different measures were identified, yet only 63 measures (of parent, child and dyadic outcomes) appeared in three or more evaluations. This level of inconsistency undermines efforts to establish the comparative effectiveness and cost-effectiveness of programs designed with similar objectives. Search 2 identified development and validation studies of identified measures. We had hoped to determine which of these measures was the most psychometrically robust and therefore eligible to be included in a small battery of recommended instruments. However, from the 18 parent outcome measures reviewed (three measuring parenting attitudes and beliefs, three measuring parenting practices, six measuring general psychological wellbeing, two measuring parent stress, four measuring parent depression), there is not one clear measure that we can definitively recommend for each outcome domain to form a core battery. This is consistent with our reviews assessing child outcome measures (see Gridley et al. 2019a) and dyadic measures (see Gridley et al. 2019b). Nevertheless, we have highlighted five parent outcome measures (one from each outcome domain) that perform comparatively well in their respective outcome domains; the PSoC (parenting attitudes and beliefs), the Parenting Scale (parenting practices), GHQ-12 (general psychological wellbeing), PSI-SF (parenting stress) and EPDS (parental depression).

Importantly, all five measures are parent self-report questionnaires and are *available* to researchers and practitioners, along with their scoring instructions, at cost from a publisher, or free from the public domain. Availability, along with other factors such as the number of items, and training and skills required to administer and score measures influence acceptability of instruments as perceived by practitioners. Self-report measures are less resource intensive and easier to implement than other approaches, and it is often appropriate that outcomes are assessed from parents’ own perspectives (Wittkowski et al. 2017). However, observational methods are considered to be the gold standard for assessing the quality of parent–child interactions (Hawes and Dadds 2006) due to their objectivity, and lack of potential bias, and diagnostic interviews are considered optimal for the assessment of mental health. A key strength of this review is that the measures identified were developed independently from program developers, providing an objective assessment of program impact.

One key barrier to identifying a small battery was that our comprehensive search and review of validation and development studies highlighted generally limited assessment of the broad range of relevant measurement properties. As observed in previous studies (e.g. Lotzin et al. 2015), internal consistency, structural validity and convergent/divergent validity are the most commonly reported properties. Investigation into other key aspects of reliability and validity is limited. Given that these measures are commonly used to assess program outcomes, the lack of evidence specifically in relation to responsiveness or sensitivity to change (or indeed stability) limits the ability to draw conclusions and make confident recommendations. Further research on these properties in existing measures is critical in the search for robust measures of intervention outcomes and should arguably be a priority over and above the future development of new measures.

Where properties are reported, there are some disappointing findings, with few measures scoring highly across multiple dimensions. The standards of quality adopted in the study to assess the methodological quality (COSMIN) and findings (adapted from Terwee et al. 2011b) of development and validation papers are high and often conflict with the thresholds reported by the authors of the validation studies (i.e. authors interpret their finding more positively). Other studies have highlighted a lack of agreement in the literature around the definitions and acceptable thresholds relating to reliability and validity (Mokkink et al. 2010). The use of such checklists was challenging and some limitations arose. The higher the number of studies/papers investigating a particular property such as structural validity for a measure—the higher the likelihood that a conflicting evidence/indeterminate rating would be assigned to that property. We developed our own approach for weighting findings according to the methodological quality of studies, but this is an area that warrants further attention as the COSMIN and associated standards evolve. While these independently developed standards were developed in the medical literature and did require some adaptation for use in our study; no alternatives are currently available in the social sciences. Despite modification, our approach contributes towards standardising the synthesis of data on measurement properties, and enhances the interpretability, generalisability and replicability of our findings.

Many measures included in this review are designed to be used with parents of a wider age range of children (i.e. to include those aged 6 years and upwards). These measures may perform differently (and more effectively) for parents of older children. It is our observation that measures designed and validated for use in a given population are often implemented by researchers in other populations, yet the validity of measures may not be generalisable to other populations (e.g. to parents of different cultural backgrounds or age ranges).

A key strength of this review is the comprehensive assessment and synthesis of psychometric evidence to support outcome measures commonly used in *RCT evaluations* of programs specifically targeted at parents antenatally and in the first 5 years of a child’s life. With calls for greater consistency in the use of measures across research studies, as well as the increasing requirement for practitioners to adopt outcome measures in routine practice, it is critical that such measures are selected on the basis of their validity, reliability and practicality. We reviewed measures commonly developed and used by researchers, partly in order to build on existing consistency in the field but also because we assumed these to be the most robust measures available and most likely to be used in practice. However, further research is needed to ensure that the identified measures are valid and reliable for parents in the early years. This challenges common assumptions about the appropriateness of measures that are deemed valid and reliable merely because they are widely used in parent evaluations.

Only measures that had been used in at least three or more RCTs were eligible in this review (in order to contribute towards promoting greater consistency in the field). However, this meant that some well-known measures (such as the Patient Health Questionnaire; PHQ) were not included or critically appraised. Newer (possibly promising) measures that have not yet been widely adopted in RCTs would have been excluded. Although previous reviews have synthesised the psychometric evidence for some of the measures not reviewed here, there is a need for further research to collate this information in one format to facilitate access, reduce time inefficiencies when searching for such information and to ensure that researchers and practitioners are consistently adopting robust measures to measure change. A limitation of this review was the exclusion of manuscripts that could not be accessed in full text format via the authors’ institutional subscriptions. This pragmatic decision meant 81 manuscripts were excluded (see Fig. 2), which could have contained useful information.

The use of RCT methodology as a proxy indicator of evaluation/measure quality may be questionable. Randomisation in and of itself does not guarantee the absence of bias, and consideration was not made of sample size and other aspects of research design such as blinding. Nevertheless, our aim was to identify the most common tools used in ‘gold standard’ RCTs, assuming the administration of the most robust measures to explore intervention effects. The list of outcomes and associated measures identified across all three reviews could form the basis for a consensus study using Delphi methods as recommended by Williamson et al. (2012) involving a range of stakeholders to generate a core outcome set.

Measures selection is challenging for practitioners and others involved in the real-world delivery, monitoring or evaluation of parenting programs. Resources and funding for child and family services are decreasing, whilst demand for evidence of impact through family outcomes is increasing (Roberts et al. 2013). We have identified promising measures that could be adopted both in research and practice to assess parent outcomes. Further research is needed to validate and test these measures for the population of interest, and further evidence synthesis is required before a consensus can be reached on a core set of measures appropriate for the evaluation parenting programs. It is important to strive for this because current levels of inconsistency in measures limits the comparability of studies and interventions and complicates messages for policy-makers and practitioners.

## Electronic supplementary material

Below is the link to the electronic supplementary material.

Supplementary material 1 (DOCX 41 KB)
