# Supplementary material for: Psychometric Properties of Parent Outcome Measures Used in RCTs of Antenatal and Early Years Parent Programs: A Systematic Review
Source: Clin Child Fam Psychol Rev. 2019 Feb 22;22(3):367–87. doi: 10.1007/s10567-019-00276-2 (PMC6669247; doi:10.1007/s10567-019-00276-2)
Supplement: Supplementary file 1 — Supplementary material 1 (DOCX 41 KB) [file 10567_2019_276_MOESM1_ESM.docx]

**Psychometric Properties of Parent Outcome Measures used in RCTs of Antenatal and Early Years Parent Programs: A Systematic Review**

**Clinical Child and Family Psychology Review**

Sarah L. Blower, Nicole Gridley, Abby Dunn, Tracey Bywater and Zoe Hindson

Department of Health Sciences, University of York

Maria Bryant

Leeds Institute of Clinical Trials Research, University of Leeds

Correspondence concerning this article should be addressed to Sarah Blower, Department of Health Sciences, University of York, York, UK, YO10 5DD. E-mail: [sarah.blower@york.ac.uk](mailto:sarah.blower@york.ac.uk)

**An Example of the Search Strategy Used For Retrieving Relevant RCT Evaluations**

Search terms included keywords related to (a) parent programmes; (b) children aged 0-5 years; and (c) evaluation studies.

*a) parent training or parent program or parent education or parent intervention.*

*b) toddler or infant or preschool or baby or child or pregnancy or antenatal.*

*c) experimental or randomised controlled trial.*

**An Example of the Search Strategy Used For Retrieving Relevant Development and Validation Studies**

**Search terms**

1. *The title of the instrument OR the associated acronym*
2. *Measurement properties*

**Worked example**

(Parenting Scale) AND (instrumentation.sh. OR methods.sh. OR Validation Studies.pt. OR Comparative Study.pt. OR psychometrics/ OR psychometry*.ab,ti. OR clinometer*.tw. OR clinometer*.tw. OR Outcome Assessment OR outcome assessment.ab,ti. OR outcome measure*.tw. OR observer variation/ OR observer variation.ab,ti. OR Health Status Indicators/ OR reproducibility of results/ OR reproducible*.ab,ti. OR discriminant analysis/ OR reliab*.ab,ti. OR unreliable*.ab,ti. OR valid*.ab,ti. OR coefficient.ab,ti. OR homogeneity.ab,ti. OR homogeneous.ab,ti. OR internal consistency.ab,ti. OR (cronbach*.ab,ti. AND (alpha.ab,ti. OR alphas.ab,ti.)) OR (item.ab,ti. AND (correlation*.ab,ti. OR selection*.ab,ti. OR reduction*.ab,ti.)) OR agreement.ab,ti. OR precision.ab,ti. OR imprecision.ab,ti. OR precise values.ab,ti. OR test-retest.ab,ti. OR (test.ab,ti. AND retest.ab,ti.) OR (reliab*.ab,ti. AND (test.ab,ti. OR retest.ab,ti.)) OR stability.ab,ti. OR interrater.ab,ti. OR interpreter.ab,ti. OR intrarater.ab,ti. OR intra-rater.ab,ti. OR intertester.ab,ti. OR inter-tester.ab,ti. OR intratester.ab,ti. OR intra-tester.ab,ti. OR interobserver.ab,ti. OR inter-observer.ab,ti. OR intraobserver.ab,ti. OR intraobserver.ab,ti. OR intertechnician.ab,ti. OR inter-technician.ab,ti. OR intratechnician.ab,ti. OR intra-technician.ab,ti. OR interexaminer.ab,ti. OR inter-examiner.ab,ti. OR intraexaminer.ab,ti. OR intraexaminer. ab,ti. OR interassay.ab,ti. OR inter-assay.ab,ti. OR intraassay.ab,ti. OR intra-assay.ab,ti. OR interindividual.ab,ti. OR inter-individual.ab,ti. OR intraindividual.ab,ti. OR intra-individual.ab,ti. OR interparticipant.ab,ti. OR inter-participant.ab,ti. OR intraparticipant.ab,ti. OR intra-participant.ab,ti. OR kappa.ab,ti. OR kappas.ab,ti. OR repeatable*.ab,ti. OR ((replica*.ab,ti. OR repeated.ab,ti.) AND (measure.ab,ti. OR measures.ab,ti. OR findings.ab,ti. OR result.ab,ti. OR results.ab,ti. OR test.ab,ti. OR tests.ab,ti.)) OR generalize*.ab,ti. OR generalist*.ab,ti. OR concordance.ab,ti. OR (intraclass.ab,ti. AND correlation*.ab,ti.) OR discriminative.ab,ti. OR known group.ab,ti. OR factor analysis.ab,ti. OR factor analyses.ab,ti. OR dimension*.ab,ti. OR subscale*.ab,ti. OR (multitrait.ab,ti. AND scaling.ab,ti. AND (analysis.ab,ti. OR analyses.ab,ti.)) OR item discriminant.ab,ti. OR interscale correlation*.ab,ti. OR error.ab,ti. OR errors.ab,ti. OR individual variability.ab,ti. OR (variability.ab,ti. AND (analysis.ab,ti. OR values.ab,ti.)) OR (uncertainty.ab,ti. AND (measurement.ab,ti. OR measuring.ab,ti.)) OR standard error of measurement.ab,ti. OR sensitive*.ab,ti. OR responsive*.ab,ti. OR ((minimal.ab,ti. OR minimally.ab,ti. OR clinical.ab,ti. OR clinically.ab,ti.) AND (important.ab,ti. OR significant.ab,ti. OR detectable.ab,ti.) AND (change.ab,ti. OR difference.ab,ti.)) OR (small*.ab,ti. AND (real.ab,ti. OR detectable.ab,ti.) AND (change.ab,ti. OR difference.ab,ti.)) OR meaningful change.ab,ti. OR ceiling effect.ab,ti. OR floor effect.ab,ti. OR Item response model.ab,ti. OR IRT.ab,ti. OR rausch.ab,ti. OR Differential item functioning.ab,ti. OR DIF.ab,ti. OR computer adaptive testing.ab,ti. OR item bank.ab,ti. OR cross-cultural equivalence.ab,ti.)

Online resource Table 2

*Criteria for good measurement properties*

| Property | Rating | Criteria |
| --- | --- | --- |
| Internal consistency | + | Cronbach alpha(s) > 0.70 OR Person Separation Index > 0.70 for IRT methods |
|  | ? | Cronbach alpha(s) not determined OR dimensionality unknown OR conflicting evidence OR Person Separation Index not determined (for IRT methods) |
|  | - | Cronbach alpha(s) < 0.70 OR Person Separation Index <0.70 for IRT methods |
| Measurement error | + | MIC > SDC OR MIC outside the LOA |
|  | ? | MIC not defined |
|  | - | MIC < SDC OR MIC equals or inside LOA |
| Reliability (inter-rater and test-retest) | + | ICC/weighted Kappa > 0.70 OR Pearson’s r > 0.80 |
|  | ? | Neither ICC/weighted Kappa or Pearson’s r calculated |
|  | - | ICC/weighted Kappa > 0.70 OR Pearson’s r > 0.80 |
| Content validity | + | All items are relevant for the construct to be measured, target population, and purpose of the measurement AND the questionnaire is considered to be comprehensive |
|  | ? | Not enough information available |
|  | - | Not all items are relevant OR the questionnaire is not considered to be comprehensive |
| Structural validity (CTT methods) | + | Factors explain > 50% of the variance OR CFI or TLI or comparable measure > 0.95 AND (RMSEA < 0.06 OR SRMR < 0.08) |
|  | ? | Not all information for ‘+’ reported |
|  | - | Criteria for ‘+’ not met |
| Structural validity (IRT methods) | + | Residual correlations among the items after controlling for the dominant factor < 0.20 OR Q3’s < 0/37, item scalability > 0.30, IRT model fit: G2 > 0.01, no DIF for important subject characteristics (such as age, gender, education): McFadden’s R2 < 0/02, OR no non-uniform DIF |
|  | ? | Important statistics not reported |
|  | - | Criteria for ‘+’ not met |
| Hypothesis Testing (convergent/ divergent validity) | + | Correlations with instruments measuring the same construct > 0.50 OR at least 75% of the results are in accordance with the hypotheses AND correlation with related constructs is higher than with unrelated constructs |
|  | ? | Solely correlations determined with unrelated constructs |
|  | - | Correlations with instruments measuring the same construct <0.50 OR < 75% of the results are in accordance with the hypotheses OR correlation with related constructs is higher than with unrelated constructs |
| Hypothesis Testing (discriminant/known groups) | + | Differences in scores on the measurement instrument for all evaluated patient subgroups are statistically significant OR > 75% of results in accordance with hypotheses |
|  | ? | Some differences statistically significant, others not |
|  | - | Differences in scores for all evaluated patient subgroups are not statistically significant OR < 75% of results in accordance with hypotheses |
| Criterion validity | + | Convincing arguments that gold standard is “gold” AND correlation with gold standard > 0.70 OR (sensitivity AND specificity > 70%) |
|  | ? | Not all information for ‘+’ reported |
|  | - | Criteria for ‘+’ not met |
| Responsive-ness | + | SDC or SDC < MIC OR MIC outside the LOA OR RR > 1.96 OR AUC ≥ 0.70; |
|  | ? | Doubtful design or method |
|  | - | SDC or SDC ≥ MIC OR MIC equals or inside LOA OR RR ≤ 1.96 OR AUC < 0.70, despite adequate design and methods |

Online resource Table 2

*Sample characteristics of studies included in the appraisal of measurement properties*

| **Measure** | **Citation**  **Country** | ***N*** | **Parent Age in years (*SD*)** | **Child Age (*SD*)** | **% Female (Parent)** | **% Female (Child)** | **Predominant Ethnicity (%)** | **Study**  **setting** | **Recruitment methods** |
| --- | --- | --- | --- | --- | --- | --- | --- | --- | --- |
| ***Parenting Attitudes & Beliefs*** | | | | | | | | | |
| **AAPI-2** | Conners et al. (2006)  Arkansus, USA | 309 | 21-30 | 48.5 months (6.3) | 92 | 52 | Caucasian (88) | General population/  Community  (Head Start) | Convenience |
| **PSoC** | Lovejoy, Verda & Hays (2010)  Illinois, USA | 91 | Range 21-49 | 4.3 years (Range 1-17) | 100 | NK | Caucasian (79%) | General population/  Community (Pre-schools) | Convenience |
| **PSoC** | Rogers & Matthews (2004)  Victoria, Australia | 849 mothers and 329 fathers | Range 18-50 | 4.9 years (Range 6 months-15 years) | 50 | 42 | NK | General population/Community (Parenting programmes) | Convenience |
| **PSoC** | Karp, Lutenbacher & Wallston (2015) Unknown, USA | 211 and 67 = 278 overall | Study 1: 27.74 (5.36); Study 2: 19.48 (1.45) | 2-12 months | 100 | NK | NK | General population/Community - Secondary analysis of hospital admission data | Convenience |
| **RSES** | Robinson et al. (2008)  Arizona, USA | 750 | 18.34 (1.65) | N/A | 63 | N/A | European American (75) | General population/Community - University students | Purposive and convenience |
| **RSES** | Chu-Lien Chao, Vidavoch & Green (2016)  Denver, USA | 524 | 20 | N/A | 60 | N/A | African American (100) | General population/Community - University students | Convenience |
| **RSES** | Hatcher & Hall (2009)  Kentucky, USA | 98 | 26.4 (5.6) | Range 2-6 years | 100 | N/A | African American (100) | General population/Community/Clinical | Convenience |
| **RSES** | Gray-Little, Williams & Hancock (1997)  North Carolina, USA | 1234 | NK | N/A | NK | N/A | NK | General population/Community - University students | Convenience |
| **RSES** | Corwyn (2000)  Unknown, USA | 13,294 | 13+ | NK | NK | NK | NK | General population/Community - Secondary analysis of longitudinal study | Convenience |
| **RSES** | Vispoel, Boo & Bleiler (2001)  Iowa, USA | 224 | NK | N/A | 74 | N/A | NK | General population/Community - University students | Convenience |
| **RSES** | Hyland et al. (2014)  Unknown, USA | 6082 | NK | N/A | 32 | N/A | African American (59) | General population/Community | Convenience |
| **RSES** | Donnellan, Ackerman & Brecheen (2016)  Texas, USA | 1127 | 18.31 (1.56) | N/A | 45 | N/A | Anglo (39) | General population/Community - University students | Convenience |
| **RSES** | Sinclair et al. (2010)  Various, USA | 702 | 44.7 (16.3) | N/A | 52 | N/A | Caucasian (71.5) | General population/Community - Online | Convenience to match desired sample characteristics |
| ***Psychological Wellbeing*** | | | | | | | | | |
| **BSI-18** | Houghton et al. (2012)  Ireland, UK | 763 | 22.2 (5.65) | N/A | 48 | N/A | Irish (100) | General population/Community - College students | Convenience |
| **BSI-18** | Prelow et al. (2005)  Boston, Chicargo & San Antonio, USA | 1137 | 32 (8.7) | NK | 96 | NK | Mexican (50) | General population/Community - Secondary analysis of longitudinal study | Stratified random sampling |
| **DASS-21** | Osman, et al. (2012)  San Antonio, USA | Study 1 887; Study 2 410 | Study 1 = 19 (2); Study 2 = 19 (3) | N/A | 60 | N/A | Caucasian Study 1 (73.5); Study 2 (63.2) | General population/Community - University students | Convenience |
| **DASS-21** | Henry & Crawford (2005)  Unknown, UK | 1794 | 41 (15.9) | N/A | 55 | N/A | NK | General population/Community | Convenience |
| **DASS-21** | Sinclair et al. (2012)  Washington, USA | 503 | 44.7 (16.3) | N/A | 52 | N/A | Caucasian (72) | General population/Community - Internet | Sample matching |
| **DASS-21** | Gomez, et al (2014)  Victoria, Australia | 687 | 43.75 (women 47.75; men 36.39) | N/A | 67 | N/A | NK | General population/Community | Convenience |
| **GHQ-12** | Hankins  (2008)  Various, England | 3705 | NK | NK | NK | NK | NK | General population/Community - taken from longitudinal study | Convenience |
| **GHQ-12** | Hankins  (2008)  Various, England | 3705 | NK | NK | NK | NK | NK | General population/Community - taken from longitudinal study | Convenience |
| **GHQ-12** | Banks  (1983)  Sheffield, England | 200 | 17 | N/A | 50 | N/A | NK | General population/Community - school leavers | Convenience |
| **GHQ-12** | Kalliath, O'Driscoll & Brough  (2004)  Various, New Zealand | 691 | 38 (Range 16-74) | N/A | 54 | N/A | European (84.7) | General population/Community - employees | Convenience |
| **GHQ-12** | Martin  (1999)  Sydney, Australia | 169 | 28 (11) | N/A | 61 | N/A | NK | General population/Community - University | Convenience |
| **GHQ-12** | Abubakar & Fischer  (2012)  Kisumu, Nairobi & Mombasa, Kenya | NK | Adults 34.04 (8.99), University students 21.08 (1.94) and Adolescents 16.79 (1.93) | NK | Adults 34.9; University students 71.6; Adolescents 42.6 | NK | NK | General population/Community | Convenience |
| **GHQ-12** | Doyle, Watson, Morgan & McBride  (2012)  Various, Northern Ireland | 4242 | 48.0 (18.1) | N/A | 58.9 | N/A | NK | General population/Community | NK |
| **GHQ-12** | Graetz  (1991)  Various, Australia | 8998 | 16-25 | N/A | 49.7 | NA | NK | General population/Community - taken from longitudinal study | Convenience |
| **GHQ-12** | Hu, Stewart-Brown, Twigg & Weich  (2007)  Various, England | NK | BHPS = 41.7 (16) years; HSE = 43 (16.4) years | NK | 52.7% in BHPS and 52.9% in HSE | NK | White (96.1) | General population/Community - taken from longitudinal study | Convenience |
| **GHQ-12** | Lewis & Wessely  (1990)  London, England | NK | 40 | N/A | 0.6 | N/A | NK | Outpatient | Random |
| **SCL-90-revised** | Chapman, Petrie & Vines  (2012) Kentucky, USA | 91 | 37 (7.28) | N/A | 100 | N/A | African American (100) | General population/Community | Convenience |
| **SCL-90-revised** | Martinez, Stillerman & Waldo  (2005)  New Mexico, USA | 205 | 17 -50 | N/A | 86 | N/A | Hispanic (50) | General population/Community - University students | Convenience |
| **SF-12** | Forero et al., (2013)  Various, Europe | 21,425 | 18+ | N/A | 52 | N/A | NK | General population/Community - secondary analysis | Pooled data/secondary analysis of a convenience sample of non-institutionalized adults |
| **SF-12** | Vilagut et al., (2013)  Various, Europe | 21,425 | 47.1 (29.3) | N/A | 52 | N/A | NK | General population/Community - secondary analysis | Pooled data/secondary analysis of a convenience sample of non-institutionalized adults |
| **STAI** | Maynard,Campbell, Devonish, Leon, Emmanuel & Roberti  (2010)  Cave Hill, Barbados | 415 | 25.2 | N/A | 75 | N/A | Caribbean (100) | General population/Community - University students | Convenience |
| **STAI** | Hundley, Gurney, Graham & Rennie  (1998) Aberdeen, Scotland | 217 | NK | N/A | 100 | N/A | NK | Hospital | Convenience |
| **STAI** | Vigneau & Cormier  (2008)  Toronto, Canada | 500 | 20.07 (3.23) | N/A | 68 | N/A | NK | General population/Community - University students | Convenience |
| **STAI** | Bieling, Antony & Swinson (1998) Pennsylvania, USA | 261 | Patient range 34.4-36.8; Non-clinical comparison 28.4 | N/A | Patient group range = 44-78; Non-clinical comparison = 61 | N/A | NK | General population/Community/Inpatient/Clinic | Convenience |
| ***Parent Stress*** | | | | | | | | | |
| **PPSDQ** | Callahan & Hynan  (2002) Wisconsin-Milwaukee, USA | 175 | Range 23.04-29.76 | Range 29.04-40.10 gestational age | 1 | N/A | NK | General population/Community | Convenience |
| **PPSDQ** | Quinnell & Hynan  (1999) Wisconsin-Milwaukee, USA | 142 | 18+ | Gestational age 37 weeks | 100 | NK | Caucasian (83) | General population/Community | Convenience |
| **PSI-SF** | Whiteside-Mansell, Ayoub, McKelvey, Faldowski, Hart & Shears  (2007) Unknown, USA | 2703 | 22.1 (5.5) | Range 15-37 months | 100 | 50 | European American (42) | General population/Community (Head Start) | Convenience |
| **PSI-SF** | Reitman, Currier & Stickle  (2002) Unknown, USA | 192 | 27.15 (4.18) | Range 3-5 years | 100 | 52 | African American(85) | General population/Community (Head Start) | Convenience |
| **PSI-SF** | McKelvey, Whiteside-Mansell, Faldowski, Shears, Ayoub & Hart  (2009) Unknown, USA | 696 | 29 (8.3) | 27 months (3) | 0 | NK | Caucasian (50) | General population/Community (Head Start) | Convenience |
| **PSI-SF** | Barroso, Hungerford, Dainelys, Graziano & Bagner  (2016)  Florida, USA | 23 | 29.88(5.28) | 13.52 months (1.3) | 100 | 47 | Hispanic (91) | General population/Community/Primary Care | Convenience |
| ***Parenting Practices*** | | | | | | | | | |
| **APQ-PR** | Clerkin, Halpern, Marks & Policaro  (2007)  New York, USA | 160 | NK | 4.47 years (0.62) | 82 | 40 | Caucasian (55.6) | General population/Community (pre-school) | Convenience |
| **MESQ** | Lagace-Seguin & Coplan  (2005)  Ottawa, Canada | 140 | 32 (6.0) | 56 (7.34) months | 100 | 48 | Caucasian (78.6%) | General population/Community | Convenience |
| **PS** | Rhoades & O'Leary  (2007)  New York, USA | 453 | Mothers 35.1 (5); Fathers 37 (6) | 5.4 (1.4) years (Range 3-7 years) | 50 | 51 | Caucasian (81) | General population/Community | Convenience |
| **PS** | Lorber, Xu, Smith Slep, Bulling & O'Leary  (2014)  New York, USA | Sample 1 453; Sample 2 399 | Range 35.06-41 | Sample 1 5.44 (1.47); Sample 2 6.65 (1.47) | NK | Sample 1 = 52; Sample 2 = 50 | Non-Latino White (85) | General population/Community | Convenience |
| **PS** | Arnold, O'Leary, Wolff & Acker (1993)  New York, USA | 168 | 31.2 | 24 months | 100 | NK | NK | General population/Community | NK |
| **PS** | Arney, Rogers, Baghurst, Sawyer & Prior (2008)  South Australia, Melbourne, Australia | 1656 | NK | Range 3-5 years | 100 | 47 | NK | General population/Community | Total sample is combined from two subsamples - first subsample random sampling of pre-schools and second sample convenience |
| ***Parental Depression*** | | | | | | | | | |
| **BDI-II** | Osman, Barrios, Gutierrez, Williams & bailey (2008)  Midwest, USA | Adolescents 414; Inpatients 377 | 15.77 (1.03) | N/A | 50 | N/A | Caucasian Adolescents (81.4), Inpatient (80.8) | General population/Community/Inpatient | Convenience |
| **BDI-II** | Makhubela & Mashegoane (2008)  Limpopo & Pretoria, South Africa | 919 | 21.70 (13.51) | N/A | NK | N/A | Black (63.4) | General population/Community - University | Convenience |
| **BDI-II** | Campbell, Maynard, Roberti & Emmanuel (2012)  Barbados, Caribbean | 415 | 25.2 | N/A | 75 | N/A | NK | General population/Community - University | Convenience |
| **BDI-II** | Kjaergaard, Elisabeth, Wang, Waterloo & Jorde (2014)  Tromso, Norway | 357 | 55.5 (10) | N/A | 52 | N/A | NK | Hospital | Convenience |
| **CES-D** | Nguyen, Kitner-Triolo, Evans & Zonderman (2004)  Various, USA | 2080 | Range 18-92 | N/A | Range 52-74 | N/A | Selected on the basis of African American & Caucasian | General population/Community - taken from longitudinal study | Convenience |
| **CES-D** | Orme, Reis & Herz  (1986)  Illinois, USA | 116 | 21.30 (7.16) | N/A | 89 | N/A | Caucasian (50) | General population/Community -attending parenting programme | Convenience |
| **CES-D** | Joseph & Lewis (1995)  Colerine, USA | 187 | 21.5 (3.2), Range 18-48 | N/A | 70 | N/A | NK | General population/Community - university students | Convenience |
| **CES-D** | Johnson, McLeod, Sharpe & Johnston  (2008)  Various, Canada | 3135 | 18-98 | N/A | 50 | N/A | NK | General population/Community | Stratified random sampling |
| **CES-D** | Pretorius  (1991)  Western Cape, South Africa | 450 | 24 (Range 19-53) | N/A | 68 | N/A | Black South African (100) | General population/Community - university students | Convenience |
| **CES-D** | Maloni, Park, Anthony & Musil (2005)  Cleveland, USA | 89 | 28.06 (6.7) | N/A | 100 | N/A | Caucasian (82) | Hospital | Convenience |
| **CES-D** | Edwards, Cheavens, Heiy & Cukrowicz  (2010)  Various, USA | 595 | 25.13 (10.07) | N/A | 68 | N/A | Caucasian (77) | General population/Community/Outpatient | Convenience |
| **CES-D** | Lieshout, Cleverly, Jenkins & Georgiades (2011)  Toronto, Canada | 668 | 33.01(4.94) | N/A | 100 | N/A | Non-migrant (67) | General population/Community | Convenience |
| **CES-D** | Atkins  (2014)  New Jersey, USA | 208 | 30.55 (7.08) | NK | 100 | NK | Black (100) | General population/Community | Convenience |
| **CES-D** | Skorikov & Vandervoort (2003)  San Fransico, USA | 261 | 26.9 (8.9) | N/A | NK | N/A | Caucasian (39) | General population/Community - university students | Convenience |
| **EPDS** | Boyce, Stubbs & Todd  (1993)  Sydney, Australia | NK | 28.4 (4.18) | N/A | 100 | N/A | NK | General population/Community | Convenience |
| **EPDS** | Carothers & Murray  (1990) Cambridge, England | 702 | Range 20-40 | NK | 100 | NK | NK | Hospital | Convenience |
| **EPDS** | Chaundron et al., (2010)  New York, USA | 422 | 24.5 (5.6) | Range 2 weeks-14 months | 100 | NK | African American (69.9) | Hospital | Convenience |
| **EPDS** | Cox, Chapman, Murray & Jones (1996)  Stoke on Trent, England | Non-postnatal 260, control group 260 | Non-postnatal 27.2; Postnatal 25.4 | NK | 100 | N/A | NK | General population/Community | Convenience |
| **EPDS** | Cox, Holden & Sagovsky  (1987) Edinburgh, Scotland | 84 | 26 | 3 months | 100 | NK | NK | General population/Community | Convenience |
| **EPDS** | Dennis  (2004) Vancouver, Canada | 594 | 28.5 | 1, 4 and 8 weeks postpartum | 100 | NK | Caucasian (91) | General population/Community | Convenience |
| **EPDS** | Drake, Howard & Kinsey  (2014)  Virginia, USA | 18 | Range 18-29 | N/A | 100 | N/A | White (72) | General population/Community | Convenience |
| **EPDS** | Edmondson, Psychogiou, Vlachos, Netsi & Ramchandani (2010)  Oxford & Milton Keynes, England | 192 | Mothers 33.3 (4.84); Fathers 35 (5.86) | 14.5 (3.04) weeks | 50 | NK | NK | General population/Community | Convenience |
| **EPDS** | Harris, Huckle, Thomas, Johns & Fung  (1989)  Caerphilly, Wales | 147 | 24.6 (5.5) | 6-8 weeks | 100 | NK | NK | Hospital | Convenience - drawn from a larger study |
| **EPDS** | Jomeen & Martin (2007)  Leeds, England | 148 | 28.86 (5.19) | Gestational age 14.11 weeks (2.71) | 100 | NK | NK | General population/Community | Convenience |
| **EPDS** | Kernot, Olds, Lewis & Maher (2015)  Adelaide, Australia | NK | 31.8 (4.3) | 0.6 (0.2) years | 100 | NK | NK | General population/Community | Convenience - drawn from a larger study |
| **EPDS** | King  (2012)  Wisconsin, USA | 169 | 23.74 (5.47) | 1.51 (1.42) months | 100 | NK | Black and Non-Hispanic (100) | General population/Community | Convenience |
| **EPDS** | Leverton & Elliot (2000)  London, England | 199 | NK | 6 weeks | 100 | NK | NK | Hospital | Convenience |
| **EPDS** | Logsdon, Usui & Nering  (2009)  Louisville, USA | 149 | 16 (1.10) | 4-6 weeks | 100 | NK | Caucasian (45) | General population/Community | Convenience |
| **EPDS** | Matthey  (2008)  Sydney, Australia | Women 238, Men 218 | Women 27.1 (4.2); Men 29 (4.6) | NK | 50 | NK | NK | General population/Community | Convenience |
| **EPDS** | Matthey, Valenti, Souter & Ross-Hamid  (2013)  Sydney, Australia | NK | 28.8 (5) | Gestational age 14.9 (4.6) weeks | 100 | NK | NK | General population/Community | Convenience |
| **EPDS** | Milgrom, Ericksen, Negri & Gemmill  (2005) Melbourne, Australia | 533 | 30.1 | 17.9 weeks | 100 | NK | NK | General population/Community | Convenience |
| **EPDS** | Phillips, Charles, Sharpe & Matthey  (2009)  Sydney, Australia | 309 | 31.9 (Range 17-44) | 5.4 months (Range 1 week - 12 months) | 100 | 47 | NK | Hospital | Convenience |
| **EPDS** | Small, Lumley, Yelland & Brown (2007)  Victoria, Australia | 1310 | NK | NK | 100 | NA | NK | General population/Community | Convenience |
| **EPDS** | Swalm, Brooks, Doherty, Nathan & Jacques  (2010)  Western Australia, Australia | 4706 | NK | Gestational age 26 weeks | 100 | NK | NK | Hospital | Convenience |
| **EPDS** | Thompson, Harris, Lazarus & Richards  (1998) Caerphilly, Wales | 1248 | NK | 1 day | 100 | NK | NK | Hospital | Convenience |
| **EPDS** | Tuohy & McVey (2008)  Glasgow, Scotland | 440 | 30.20 (4.52) | 6.50 (3.22) months | 100 | NK | NK | General population/Community | Convenience |
| **EPDS** | Venkatesh, Ziotnick, Triche, Ware & Phipps (2014) Massachusetts, USA | NK | 16 (Range 13-18) | 6 weeks | 100 | NK | Hispanic (52.8) | General population/Community | Convenience - drawn from a larger study |
| **HAMD** | Zimmerman, Martinez, Young & Chelminski (2013)  USA | 627 | 41.2 (12.3) | N/A | 65 | N/A | White (88.2) | Community/ Outpatients | Convenience |
